# Differentiation of self and its relationship with marital satisfaction and parenting styles in a Spanish sample of adolescents’ parents

**DOI:** 10.1371/journal.pone.0265436

**Published:** 2022-03-23

**Authors:** Marta Mozas-Alonso, Jesús Oliver, Ana Berástegui

**Affiliations:** 1 Department of Psychology, Facultad de Ciencias Humanas y Sociales, Universidad Pontificia Comillas, Madrid, Spain; 2 Department of Personality, Assessment and Psychological Treatment, Facultad de Psicología, Universidad de Málaga, Málaga, Spain; 3 Instituto Universitario de la Familia, Universidad Pontificia Comillas, Madrid, Spain; Washington University, St. Louis, UNITED STATES

## Abstract

The present study aims to test some of the assumptions of Bowen’s Family Systems Theory, specifically, to assess the relationships between differentiation of self (DoS), marital satisfaction and parenting styles, as well as the gender differences in these variables in a Spanish sample. The sample includes 140 Spanish non-single adults, over 30 years old, who have at least one adolescent son or daughter, aged between 12 and 19 years old. The instruments used were a sociodemographic questionnaire, the Differentiation of Self Scale, the Warmth Scale-Parents, the Rules and Demands Scale-Parents and the Satisfaction subscale of the Dyadic Adjustment Scale. The results show DoS is related to marital satisfaction, and parenting styles’ dimensions. Regarding gender, women show higher emotional reactivity and warmth-communication than men do. Furthermore, marital satisfaction mediates the relationship between DoS and parental warmth-communication, criticism-rejection and inductive form. Finally, implications for clinical practice and future research are discussed.

## Introduction

DoS is an essential concept of Bowen’s Family Systems Theory [1], which is defined as the degree to which one is able to balance a) emotional and intellectual functioning and b) intimacy and autonomy in relationships.

At the intrapsychic level, DoS involves the ability to distinguish thoughts from emotions and the ability to decide on which of them to act [1]. Therefore, a person with a high level of DoS is expected to be flexible, versatile and to have greater facility to manage stress, whereas a person with a low level of DoS is expected to act in an emotionally reactive way or, on the contrary, in an excessively rationalizing way [2].

In the interpersonal level, DoS implies the ability to maintain the balance between intimacy with others and the independence of them [2]. A highly differentiated person can be emotionally close to others without implying emotional fusions or losses of identity. However, a poorly differentiated person will be highly reactive to the dictates of his family, adapting to them in a submissive manner or, on the contrary, rebelling against them [1].

Skowron and Friedlander [2] and, later, Skowron and Schmitt [3] operationalized DoS into four dimensions: a) *Emotional Reactivity*: the degree to which a person responds to environmental stimuli with emotional decontrol, emotional lability or hypersensitivity; b) *I Position*: sense of self defined with clarity and ability to rationally adhere to one’s convictions, even when one is pressured to do the opposite; c) *Emotional Cutoff*: feeling of threat to privacy, as well as excessive vulnerability in relationships with others; and d) *Fusion with Others*: overinvolvement with others, including triangulation and overidentification with parents. Later, Oliver and Berástegui [4] identified a fifth dimension of DoS, congruent with Bowen’s Theory: e) *Dominance over the Others*: tendency to pressure others to adapt to their own interests, to tolerate little differences of opinion, to enter into power struggles with others and to be dogmatic.

Scientific research about DoS supports many of the basic assumptions of Bowen’s Theory [1]. Several studies have found that a greater degree of DoS is related to lower trait anxiety, fewer physical and psychological health problems, greater emotional regulation, more secure attachment in adults, and better family and marital functioning [4–10].

One of Bowen’s postulates [1, 11] indicates that most differentiated people have better couple and parent-child relationships, because they do not tend to regulate their anxiety through the couple conflicts, the emotional distancing from the partner, the under or overfunctioning, or the focus of anxiety on their children. Thus, this theory predicts that the level of parents’ DoS is associated with their marital satisfaction and parenting styles, and that marital satisfaction could mediate the relationship between parents’ DoS and their parenting style, because parents do not need to focus on a child to calm their anxiety and solve their relationship problems.

Some studies have analyzed the relationship of adult’s DoS with their marital satisfaction and their parenting style [12–15]. However, the studies are still scarce, and none that have analyzed the mediating role of marital satisfaction on the relationship between DoS and parenting styles.

### Differentiation of self and marital satisfaction

Marital satisfaction is the degree in which spouses feel good about themselves, their partner and their marriage in a subjective and regular basis [16]. Couples with higher levels of marital satisfaction in both members show lower level of stress, greater happiness with respect to their life and greater ability to face adverse living conditions [16]. Jackson et al. [17] carried out a meta-analysis with 101,110 participants concluding that there are no differences between wives and husbands in marital satisfaction along non-clinical samples.

According to Bowen [1], adults’ level of DoS relates to their marital quality and satisfaction. Specifically, couples with a lower degree of DoS have lower emotional maturity and lower capacity for intimacy and/or autonomy, while couples with a higher level of DoS are more flexible, tolerate differences better and have greater intimacy.

Research shows that a higher level of DoS predicts higher levels of marital satisfaction in American samples [18–20]. In Spain, several studies carried out by Rodríguez-González [14], also found the same result. Studies do not agree on which are the DoS dimensions related to marital adjustment. Some studies suggest that all the dimensions predict marital satisfaction [20, 21], while others find that the main dimension that predicts low marital satisfaction is emotional cutoff [14, 15].

### Differentiation of self and parenting styles

Parenting styles refer to the way that adults interact with their children in everyday situations, making decisions or resolving conflicts [22]. Baumrind [23] proposes three styles through which parents control the behavior of their children: a) authoritarian style, b) permissive style and c) authoritative or democratic style.

Later, Maccoby and Martin [24] state that two dimensions define parenting styles: warmth/communication and control/demand. *Warmth/communication* refers to the degree of sensitivity and responsiveness of parents to the needs of their children, above all, of an emotional nature; and *control/demand* refers to pressure or number of demands that parents exercise on their children to achieve certain objectives and goals. Four parenting styles emerge from the combination of these dimensions: a) authoritarian style, with low warmth/communication and high control/demand; b) permissive style, with high warmth/communication and low control/demand; c) negligent style, with low warmth/communication and low control/demand; and d) authoritative style, with high warmth/communication and high control/demand.

Nowadays, the idea that there is a single optimal parenting style is questioned, since the developmental stage or the context and culture may play an important role in it. Several studies carried out with Spanish adolescents, find that the indulgent and authoritative parenting styles relates to better psychological adjustment, greater self-concept, and fewer behavior problems in adolescents. On the while, the authoritarian style relates to a greater number of psychological symptoms, more disruptive behaviors, and greater substance abuse in adolescents [25–28].

According to Bowen’s theory [1], parents with a greater degree of DoS are expected to provide support to their children and take care of them in such a way that allow an appropriate autonomy for their evolutional stage and enhance the development of their capacities for emotional and behavioral self-regulation. Specifically, Ragelienė and Justickis [29] associate the characteristics of a low DoS in parents with the authoritarian style a) inability to understand the child’s needs and distinguish them from their own needs or desires; b) inability to accept and understand the child’s feelings and thoughts as different from their own, as well as to take them into consideration when relating to him; or c) inability to make flexible and adjust the norms to the needs of the child or to a particular situation.

Over the years, few authors have studied the relationship between the degree of parents’ DoS and their parenting styles and they have focused mainly in young children. For example, Kriščiūnaitė and Pakrosnis [30] conducted a study with 92 parents of preschool children in Lithuania, and concluded that a high degree of DoS in parents correlated positively with an authoritative parenting style, whereas it did negatively with an authoritarian style. These authors hypothesize that the poor interpersonal communication skills, characteristic of poorly differentiated people, increase the probability to resolve their conflicts in a more aggressive and authoritarian way. Gorbani and Amani [12] conducted a study, with a sample of 500 parents in Iran, which showed I position was positively related to authoritative parenting style while emotional cutoff was negatively related to. Another study, carried out by Lee and Han [13] with a sample of 411 South Korean mothers, observed that more differentiated mothers were less overprotective.

Parenting styles also show relationships with adolescents and college students’ DoS. Ragelienė and Justickis [29] found that the democratic style relates to a higher level of DoS in adolescents, while the authoritarian style relates to a lower level of DoS. Schwartz et al. [31] found a different impact depending on gender: perceiving their parents as having a disapproving parenting style was associated with a lesser I position in male students, while a lack of emotion-coaching style was associated with higher levels of fusion and lower levels of emotional cutoff in female students.

### Gender differences in differentiation of self, marital satisfaction and parenting styles

Studies about gender and DoS have provided inconsistent results, with some reporting no gender differences [32, 33] while others showing differences on some dimensions. For example, in U.S. samples men score higher in Emotional Cutoff, while women do so in Emotional Reactivity [20]. In Spanish samples, women tend to show greater emotional reactivity than men [4–7], and in an Iranian sample, male adolescents scored higher on the I Position, while female adolescents scored higher on emotional cutoff [34].

Concerning the gender differences in marital satisfaction, Jackson et al. [17] carried out a meta-analysis with 101,110 participants and they concluded that the tendency of the women to manifest less marital satisfaction than men in heterosexual couples is only significant in clinical samples while no gender differences are found in marital satisfaction in non-clinical population within and without the same couple.

Regarding the study of gender differences in parenting styles, several authors agree that children score their mothers higher than their fathers in all dimensions of parenting styles in Spain [28, 35–37]. This means that, according to the children’s perspective, mothers are warmer and more communicative and accepting than fathers, but also more controlling, authoritarian and permissive. Gender stereotypes, that still associate women with the childrearing largely than men, could explain these results. Despite these differences, the high correlations found between maternal and paternal style, indicate that children perceive the educational practices of both parents in a similar way [35, 36, 38].

### Differentiation of self, marital satisfaction, and parenting styles during adolescence

Bowen’s Theory [1] predicts that in adults with low DoS, triangulation processes between marital and parental undifferentiated relations may concur [39]. These parents may project their anxiety and over-reactivity from their couple discords into their children, and from their parenting difficulties into the couple, thus limiting the children DoS process [40].

Some empirical studies associate a positive marital relationship with better parental-child relationships [41, 42] and specifically with a sensitive, warm, and responsive parental style [43–45]. By the one hand, parents engaged in satisfied couples can transfer this affect and behavior from the marital relationship to parent-child interaction in the intrapersonal level (spill-over hypothesis). By the other hand, marital satisfaction can also stimulate parental cooperation and communication in the education of children in the interpersonal level (crossover hypothesis), and this is associated with fewer attempts to triangulate one of their children to undermine the capacity and authority of the other partner [44].

However, the relationship between DoS, marital adjustment and parenting styles has been scarce and indirectly assessed [39]. Although the relationships between these variables are supposed to be circular, we propose a mediation model as a priority effect, in which DoS affects parenting styles directly and indirectly, through marital satisfaction, combining spillover and crossover processes [44].

Moreover, the children transition into adolescence is an important stage of the family life cycle in which parental and marital relationships need change and adaptation, making adults DoS a key variable at this stage [46]. On the one hand, the changes in adolescent parenting needs and challenges can make marital satisfaction and communication an important support for this transition; and parenting stress an important challenge to marital satisfaction. On the other hand, adolescence has been described as a period of marital reunion after childrearing; the extent to which this encounter is satisfying or not can influence the parents’ ability to cope with the adolescent’s parenting. Thus, DoS can affect both marital satisfaction and parenting styles and the relationships between them, facilitating the whole family healthy transition through this life cycle stage.

Finally, it has being hypothesized that DoS may show differences in its configuration and impact between cultures [32, 47]. Therefore, the study of DoS in a Spanish sample can be useful to understand its influence in couple and parental relationships in this particular culture.

The aims of this study are: a) to confirm the relationships between DoS, marital satisfaction and parenting styles in a Spanish sample of adolescents’ parents; b) to analyze the gender differences in DoS, parenting styles and marital satisfaction; c) to test whether marital satisfaction mediates the relationship between parents’ DoS and parenting styles dimensions.

## Method

### Participants

In this study, participants over 30 years of age, of Spanish nationality, that cohabit with their couple for at least 3 years and have at least one child whose age is between 12 and 19 years old, were included. A convenience sampling was conducted, followed by a snowball procedure.

The sample obtained consists of 140 Spanish participants between 37 and 62 years old (*M* = 48.72, *SD* = 4.30), 50 men (35.7%) and 90 women (64.3%). 78.6% of the participants completed university studies, 16.4% had higher secondary education, 3.6% had lower secondary education and 1.4% had primary education. Most of them (92.9%) had a two-parent nuclear family, while the remaining 7.1% had a reconstituted family. The participants cohabited with their partners between 3 and 35 years (*M* = 20.20, *SD* = 5.64). They have between 1 and 5 children (*M* = 2.09, *SD* = 0.73). Target adolescents were between 12 and 19 years old (*M* = 15.20, *SD* = 1.97), 50.7% were male and 49.3% were female.

### Instruments

#### Sociodemographic questionnaire

This personal information self-report was constructed *ad hoc* to gather the following variables: sex, age, level of education, type of family of origin (two-parent, single-parent, reconstituted), number of years of cohabitation with the couple, number of children and age of the target adolescent.

#### Differentiation of Self Scale (DSS; in Spanish, EDS) [4]

This instrument assesses the intrapsychic and interpersonal dimensions of DoS in adults. It consists on a 74 items Likert scale with six response options (from 1 -strongly disagree- to 6 -very much agree-) and five factors: a) I Position (IP), b) Emotional Reactivity (ER), c) Fusion with Others (FO), d) Dominance over the Others (DO) and e) Emotional Cutoff (EC). The first subscale has a direct relationship with the construct, that is, the higher the IP, the higher the degree of DoS. The last four subscales have an inverse relationship with the construct, with higher scores reflecting a correspondingly lower degree of DoS. The total score of each subscale, as well as the full scale, is between 1 and 6. The original scale presents good reliability and validity reports. The Cronbach’s Alpha coefficients of the scale and the subscales indicate a high internal consistency: *α* = .93 in total DSS, *α* = .86 in IP, *α* = .89 in ER, *α* = .90 in FO, *α* = .89 in DO, and *α* = .90 in EC. In this study, the scales also show high internal consistency: *α* = .87 in total DSS; *α* = .75 in IP; *α* = .84 in ER; *α* = .83 in FO; *α* = .89 in DO; and *α* = .84 in EC.

#### Satisfaction subscale of Dyadic Adjustment Scale (DAS) [48]

This instrument assesses the quality of the couple’s relationship through four subscales and a total score of Dyadic adjustment. In the present study, only the Satisfaction subscale was used, which consists of a 10 items Likert scale (with variable range according to the question). In the present study, the typified scores were used with a score rank between 20 and 80. The internal consistency of Satisfaction subscale is high: *α* = .88 and the internal consistency index of the subscale obtained in this study was high: *α* = .86.

#### Warmth Scale-Parents and Rules and Demands Scale-Parents (WS-P and RDS-P; in Spanish: EA-P and ENE-P) [49]

These instruments assess self-reported parenting styles. Parents are asked to respond focusing on one target adolescent son or daughter. The Warmth Scale consists of 20 items Likert scale with five options (from 1 -Never- to 5-Always-) and two factors: a) *warmth-communication* and b) *criticism-rejection* of parents towards their children. The total score for each factor ranks between 10 and 50. The Rules and Demands Scale is a 28 item Likert scale with five options (from 1 -Never- to 5-Always-) composed of three factors that follow the model of Baumrind [23]: a) *inductive form*, i.e., the tendency to explain the rules to the children, and adapt them to the needs and possibilities of the children; b) *rigid form*, i.e., the tendency to impose the compliance with the rules on the children, and maintain a level of demand that is too high and inappropriate to the needs of the children; and c) *indulgent form*, i.e., the tendency not to set rules or limits to the children’s behavior, and if it is done, not to demand compliance. The total score ranked between 10 and 50 for the first two factors and between 8 and 40 for the third. The internal consistency indices of these scales are: *α* = .78 in Warmth-Communication, *α* = .66 in Criticism-Rejection, *α* = .68 in Inductive form, *α* = .68 in Rigid form and *α* = .60 in Indulgent form [50]. The original scale shows good validity reports. In the present study, internal consistency indices indicated a good discriminative capacity: *α* = .84 in Warmth-Communication; *α* = .80 in Criticism-Rejection; *α* = .81 in Inductive form; *α* = .74 in the Rigid form, and *α* = .76 in the Indulgent form.

### Procedure

The study participants accessed an online survey through Google Form that was disseminated with a convenience sampling through social networks. Participants were asked to share the study with their contacts. The form explained the research aims, the inclusion criteria and the instructions to complete the questionnaire. It also guaranteed the anonymity of the answers and the protection of the data, and appreciated the participation and dissemination of the study. The universal ethical principles governing the conduct of research in psychology have been respected, including maintaining confidentiality and obtaining informed consent from participants. Data from people who met all the inclusion criteria were entered into SPSS-24.

### Data analysis

To determine the appropriate statistical test in each case, the assumptions for each of the tests were checked. The Pearson’s r test was used to analyze the relationships between variables. Furthermore, the Student’s t for independent samples was used to test differences of means. The effect size indices used according to the statistical test were: Pearson’s correlation coefficient (*r*), and the Cohen’s *d* for Student’s t. Moreover, the mediation analysis was performed, using a bootstrapping procedure [51] with the macro PROCESS version 3 for SPSS [52], to check if marital satisfaction mediates the effect of DoS on the dimensions of parenting styles, including gender as a covariate. The parameter estimates were based on 10,000 bootstrap samples. The mediational model 4 was used; likewise, confidence interval for the indirect effect was analyzed, and it was considered that if the interval did not include zero it would indicate a statistically significant indirect effect with *p* < .05 [53].

## Results

### Correlations between differentiation of self, marital satisfaction and parenting styles

Table 1 shows the relationships between DoS, marital satisfaction and parenting styles. The results indicated moderate positive relationships between DoS and Marital satisfaction (*r*^*2*^ = .10). Also, there is a negative relationship between Emotional Cutoff and Marital satisfaction, with moderate effect size (*r*^*2*^ = .18).

**Table 1:** Matrix correlations between differentiation of self, parenting styles dimensions and marital satisfaction.

	Marital Satisfaction	Warmth-Communication	Criticism-Rejection	Inductive form	Rigid form	Indulgent form
DSS	.32**	.30**	-.32**	.31**	-.32**	-.27**
IP	.13	.36**	-.28**	.37**	-.08	-.17*
ER	-.11	.05	.20*	-.10	.20*	.02
FO	-.13	-.26**	.19*	-.24**	.14	.26**
DO	-.19*	-.17*	.14	-.14	.29**	.17*
EC	-.43**	-.31**	.22*	-.20*	.25**	.25**
Marital Satisfaction	*---*	.39**	-.40**	.29**	-.15	-.10

*Note*. DSS = Differentiation of Self Scale; IP = I Position; ER = Emotional Reactivity; FO = Fusion with Others; DO = Dominance over the Others; EC = Emotional Cutoff.

*p < .05

**p < .01.

DoS was positively related to Warmth-Communication (*r*^*2*^ = .09) and the Inductive form (*r*^*2*^ = .10), both with a moderate effect size. They also showed negative relationships between DoS and Criticism-Rejection (*r*^*2*^ = .10), the Rigid form (*r*^*2*^ = .10) and the Indulgent form (*r*^*2*^ = .07), all of them with a moderate effect size. Moderate significant relationships were also found between the DoS dimensions and the parenting styles. Specifically, Warmth-Communication was positively related to I position (*r*^*2*^ = .13) and negatively related to Emotional Cutoff (*r*^*2*^ = .10) and Fusion with Others (*r*^*2*^ = .07); furthermore, Criticism-Rejection was negatively related to I Position (*r*^*2*^ = .08). On the other hand, the Inductive form was positively associated with I Position (*r*^*2*^ = .14) and Fusion with Others (*r*^*2*^ = .06), while the Rigid form was positively related to Dominance over the Others (*r*^*2*^ = .08), and the Indulgent form was positively related to Fusion with Others (*r*^*2*^ = .07) and Emotional Cutoff (*r*^*2*^ = .06).

On the contrary, the rigid form was positively associated with dominance over the others, emotional cutoff and emotional reactivity, while the indulgent form was positively associated with fusion with others, emotional cutoff and dominance over the others, and negatively with I position

Finally, Marital satisfaction is positively and moderately related to Warmth-Communication (*r*^*2*^ = .15) and the Inductive form (*r*^*2*^ = .08); and negatively and moderately related to Criticism-Rejection (*r*^*2*^ = .16). However, no significant relationships were found between Marital satisfaction and the Rigid or the Indulgent form.

### Gender differences in differentiation of self, marital satisfaction and parenting styles

Table 2 presents the differences found in DoS, parenting styles dimensions and marital satisfaction according to gender. Significant differences were observed between men and women in Emotional Reactivity scores, with a large effect size, being higher the scores of women than those of men. However, no significant differences were found in any other dimensions of DoS. Furthermore, significant differences were observed between men and women in Warmth-Communication, with a small effect size, and with women having higher scores than men. Transmission of norms, expression of affection and marital satisfaction, did not show significant gender differences either.

**Table 2:** Mean differences between men and women in differentiation of self dimensions, marital satisfaction and parenting styles dimensions.

		Men		Women				
		*Mean*	*S*.*D*.	*Mean*	*S*.*D*.	*t*_*(gl)*_	*p*	*d*
Differentiation of Self	DSS	4.36	0.41	4.23	0.45	-1.587_(138)_	.115	.28
	IP	4.66	0.49	4.67	0.54	0.121_(138)_	.904	.02
	ER	2.88	0.65	3.56	0.87	5.25_(126)_**	< .001	.85
	FO	2.61	0.67	2.75	0.72	1.18_(138)_	.240	.21
	DO	2.75	0.73	2.66	0.85	-0.56_(138)_	.573	.10
	EC	2.65	0.56	2.52	0.68	-1.08_(138)_	.282	.19
Marital Satisfaction	Satisfaction	46.24	11.89	46.49	11.54	0.12_(138)_	.904	.02
Parenting Styles dimensions	Warmth-Commun.	43.14	5.14	44.74	4.25	1.98_(138)_*	.049	.35
	Criticism-Reject.	17.50	5.44	18.58	4.49	1.26_(138)_	.210	.22
	Inductive form	43.66	3.86	43.41	4.68	0.32_(138)_	.749	.13
	Rigid form	25.76	6.03	27.22	5.37	1.48_(138)_	.142	.26
	Indulgent form	15.14	4.17	14.58	4.32	0.75_(138)_	.457	.06

*Note*. DSS = Differentiation of Self Scale; EC = Emotional Cutoff; FO = Fusion with Others; ER = Emotional Reactivity; DO = Dominance over the Others, IP = I Position. Criticism-Reject. = Criticism-Rejection; Warmth-Commun. = Warmth-Communication.

*p < .05

**p < .01.

### Mediation analysis

To explore whether marital satisfaction mediated the relationship between DoS and its parenting styles related dimensions (warmth-communication, criticism-rejection and inductive form) three simple mediation analyses were carried out, including gender as a covariate. Mediation analyses were not carried out with rigid and indulgent forms, because the assumption of linear relationship between marital satisfaction and these variables were not met [52].

**Fig 1:**
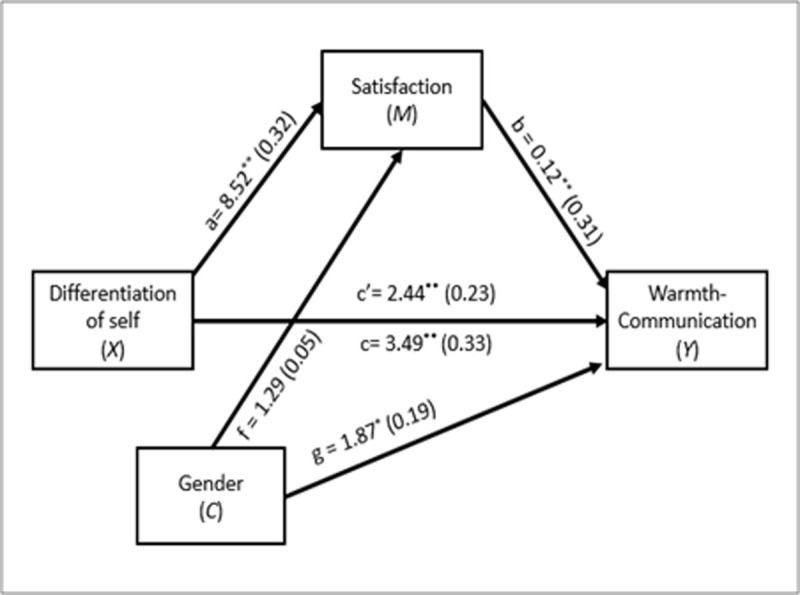
First mediation model. c = total effect of *X* on *Y*; c’ = direct effect of *X* on *Y*; a = effect of *X* on *M*; b = effect of M on *Y*; f = effect of *C* on *M*; g = effect of *C* on *Y*. Values in parentheses represent standardized coefficients.*p < .05; **p < .01.

As shown in Fig 1, the first mediation analysis revealed that there was a total effect of DoS on Warmth-Communication (*B =* 3.49, *Beta* = .33, *SE =* .85, *t*(137) = 4.13, *p* < .001), with a direct effect (*B =* 2.44, *Beta* = .23, *SE =* .85, *t*(136) = 2.87, *p* = .005), and an indirect effect via Marital satisfaction (*B =* 1.06, *Beta* = .10, *SE =* .41, 95% *CI* [.34, 1.95]). These results indicate that a higher level of DoS is related to a greater warmth-communication, and a significant proportion of this relation is explained by the mediating role of marital satisfaction.

**Fig 2:**
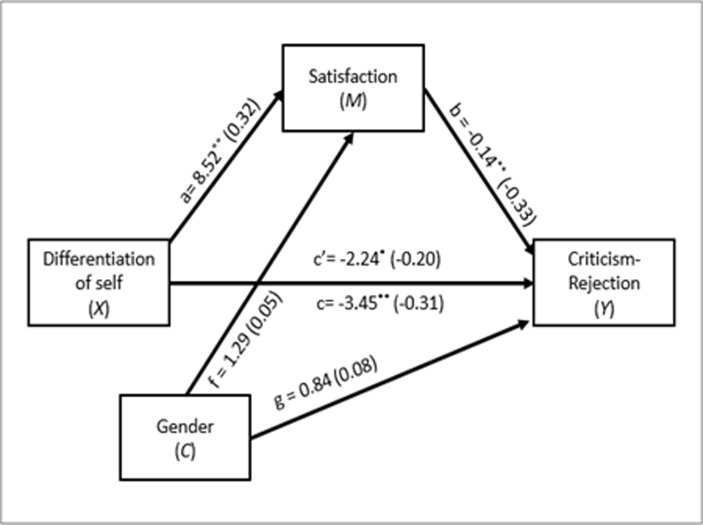
Second mediation model. c = total effect of *X* on *Y*; c’ = direct effect of *X* on *Y*; a = effect of *X* on *M*; b = effect of *M* on *Y*; f = effect of *C* on *M*; g = effect of *C* on *Y*. Values in parentheses represent standardized coefficients. **p < .05; **p < .01.

As Fig 2 shows, a second mediation analysis revealed that there was a total effect of DoS on Criticism-Rejection (*B =* -3.45, *Beta* = -.31, *SE =* .90, *t*(137) = -3.83, *p* < .001), with a direct effect (*B = -*2.24, *Beta* = -.20, *SE =* .90, *t*(136) = -2.50, *p* = .014), and an indirect effect via Marital satisfaction (*B =* -1.21, *Beta* = -.11, *SE =* .49, 95% *CI* [-2.27, -0.38]). These results show that a higher level of DoS is related to a lower criticism-rejection, and a significant proportion of this relation is explained by the mediating role of marital satisfaction.

**Fig 3:**
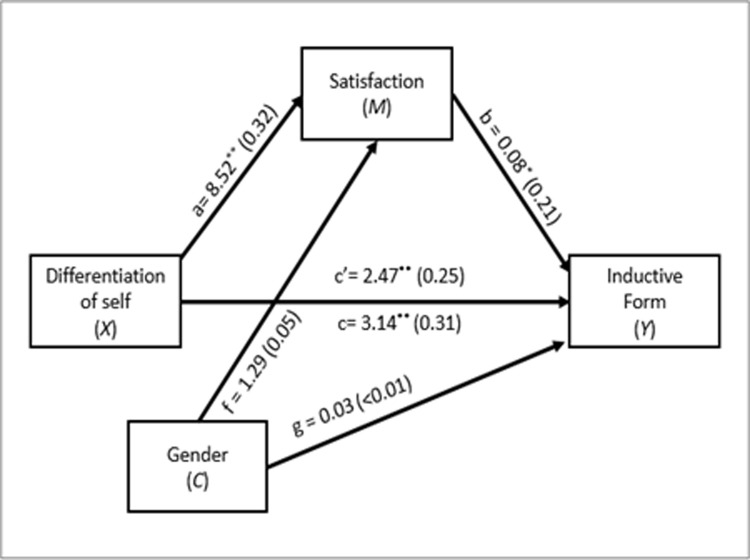
Third mediation model. c = total effect of *X* on *Y*; c’ = direct effect of *X* on *Y*; a = effect of *X* on *M*; b = effect of *M* on *Y*; f = effect of *C* on *M*; g = effect of *C* on *Y*. Values in parentheses represent standardized coefficients. **p < .05; **p < .01.

Finally, as shown in Fig 3, the third mediation analysis also revealed that there was a significant total effect of DoS on Inductive form (*B =* 3.14, *Beta* = .31, *SE =* .82, *t*(137) = 3.84, *p* < .001), with a direct effect (*B =* 2.47, *Beta* = .25, *SE =* .85, *t*(136) = 2.91, *p* = .004), and an indirect effect via Marital satisfaction (*B =* .68, *Beta* = .07, *SE =* .28, 95% *CI* [.16, 1.26]). These results show that a higher level of DoS is related to a greater inductive form, and a significant proportion of this relation is explained by the mediating role of marital satisfaction.

## Discussion

In the present study, some of the assumptions of Bowen’s Family Systems Theory [1] were tested in a Spanish sample. Specifically, the relationships between DoS, parenting styles and marital satisfaction were studied. In addition to this, the differences according to gender in these three variables were analyzed.

The results support that there is a positive relationship between DoS and marital satisfaction. These results are consistent with Bowen’s postulate [1] which states that the most differentiated people have more satisfactory relationships, and also with several US studies that conclude that a higher level of DoS predicts higher levels of marital satisfaction [18, 19], as well as with some studies conducted in Spain on the relationship between these two variables [14].

Moreover, the significant negative relationship found between emotional cutoff and satisfaction in the couple, is consistent with Rodríguez-González et al. [14, 15]. This can be explained by the difficulty that these people have with intimacy, as it is one of the main components of couple relationships. In this way, physical or emotional distancing could put at risk the dialogue, the affection, the commitment, the ability to resolve the conflicts of the couple and, ultimately, the marital satisfaction. Moreover, in the present study there is also a negative relationship between marital satisfaction and dominance over the others. This relationship may be due to the difficulty that these people have to tolerate differences of opinion, being common symmetrical escalations in the couple. Given that the validation of the scale that includes this last dimension has recently been published [4], there is no previous empirical evidence about this relationship.

Regarding DoS and parenting styles, as expected, parents’ DoS was positively related to the inductive form of transmitting the rules to adolescents and with the expression of affection and communication towards them. Likewise, DoS was negatively related with the rigid and indulgent forms of transmission of norms and with the expression of criticism and rejection to the children. These results are consistent with those of Kriščiūnaitė and Pakrosnis [30], Gorbani and Amani [12], and Lee and Han [13], who observed that DoS is positively related to the authoritative style and negatively related to the authoritarian or overprotective styles.

Moreover, it was found that warmth-communication was positively related to I position and negatively related to emotional cutoff, fusion with others and dominance over the others; likewise, the criticism-rejection was negatively related to I position and positively related to emotional reactivity, emotional cutoff and fusion with others. It was also observed that the inductive form was positively related to I position and negatively related to emotional cutoff and fusion with others. On the contrary, the rigid form was positively associated with dominance over the others, emotional cutoff and emotional reactivity, while the indulgent form was positively associated with fusion with others, emotional cutoff and dominance over the others, and negatively with I position. These results agree with those of Gorbani and Amani [12], who also found that the indulgent form was positively related to I position and negatively related to emotional cutoff, the rigid form was positively related to emotional cutoff, and the indulgent form was associated with fusion with others. Likewise, Lee and Han [13] observed that overprotection was negatively related to I position and positively related to fusion with others, emotional cutoff and emotional reactivity.

The relationships found between the dimensions of DoS and warmth-communication and criticism-rejection can be explained by the following [1, 11]. First, people with higher I position can feel more secure to show their affection and to communicate with their children. Secondly, people with greater emotional cutoff may have fewer warmth expressions and communication with their children to avoid intimacy, using criticism and rejection to maintain physical or emotional distance with them. In third place, people with higher scores in fusion with others may be less affectionate and communicative with their children since they feel fear of being rejected if they express what they really think and, on the other hand, can show criticism or rejection attitudes towards their children when their children move away from them or try to differentiate themselves. In fourth place, people with greater emotional reactivity can show more signs of criticism and rejection towards their children because they respond to stimuli in an autonomous and uncontrolled way. Finally, people with higher scores in dominance over the others may be less warm and communicative with their children since they do not usually take into account the interests of others [4].

One possible explanation of the relationship between DoS and the inductive form may be that more differentiated parents have a more defined conception of the own self and a higher ability to distinguish between one’s own thoughts and feelings and those of the others [1, 2]. This may favor the two-way communication between parents and children, because these parents will tend to take into account the interests of their adolescents and, in turn, direct their behavior through reasoning and negotiation [11]. In addition, parents with a higher degree of DoS are less emotionally reactive, so it would be expected that in times of stress, like adolescence, they would be less likely to overreact, overprotect, criticize or reject their adolescents.

Instead, the difficulty of regulating one’s own emotions, understanding the needs of adolescents and distinguishing them from their own, as well as the tendency to relate to others in a dominant way, can be associated with an authoritarian style, in which parents impose the norms to their children without considering their interests [11, 23, 24]. This is consistent with the lack of warmth and communication expressed by parents with these characteristics. According to the results, these parents could respond in a labile and uncontrolled way to situations in which their adolescents behave contrary to what they expect, or show some autonomy, even using physical or emotional distance. In these cases, parents with a rigid style are likely to express criticism and rejection to their children.

On the other hand, a more indulgent educational style can be characteristic of parents who tend to emotional fusion with others, that is, they renounce their own criteria to avoid conflicts and get approval from others [1, 23, 24]. However, in view of the results, there may be times when these parents try to redirect the situation by adopting dominant attitudes or moving away from conflict situations. In any of these three cases, the warmth expression and communication towards the children are low while the expressions of criticism and rejection are high.

The tests carried out between marital satisfaction and parenting styles confirm that marital satisfaction correlates positively with the inductive form of transmitting the rules and with the warmth expression and communication, while it does in a negative way with the criticism and rejection. As Pedro et al. [44] point out, couples with greater marital satisfaction may be more able to cooperate in the education of their children, being less likely to triangulate any of them. In this way, both parents can adopt a more inductive style, without any of them having to assume, for example, a more indulgent role to coalesce with one of their children against the other member of the couple. It seems more likely that spouses who prefer direct communication and warmth can repeat these same patterns in their relationship with their children, while a triangulating couple tend to express criticism and rejection towards their children, either by the member against whom the child is coalesced or by both parents when dealing with an attacking deviating triad.

In contrast with the literature, the results did not find a relationship between marital satisfaction and rigid and indulgent forms of transmitting the rules to adolescents [43, 45]. Parenting styles degree of agreement or disagreement within the couple may have a differential impact on marital satisfaction [54], that is, the greater the agreement, the greater the satisfaction. Future studies that assess parenting styles in both members of the couple could clarify the nature of this relationship.

When gender differences were analyzed, women presented a greater emotional reactivity than men, which coincides with the results of several studies in Spanish samples [4–7] (e.g.,. However, the differences regarding the emotional cutoff dimension found in other studies were not observed [4]. The limited number of men in this study should make us take these results with caution. Secondly, no gender differences were found in marital satisfaction, consistently with previous literature in non-clinical population [17]. Finally, women scored higher in warmth-communication than men, but no gender differences were observed in the other dimensions of parenting styles. These results agree with Etxebarría et al. [55], who also found, in a sample of 3,711 Spanish parents with children, that mothers were warmer and more unconditionally accepting than men. García-Moral et al. [35] Mendo et al. [56] and Oliva et al. [36] also observed that Spanish adolescents perceived mothers as warmer and more communicative than fathers. Furthermore, García-Moral et al. [35], Horvath et al. [38] or Oliva et al. [36] found a great concordance between parental and maternal parenting styles. However, Mendo et al. [56] found that women use more inductive and rigid forms, and García-Moral et al. [35] and Oliva et al. [36] observed that women controlled their children more than men.

Finally, results revealed that marital satisfaction mediates the relationship between DoS and warmth-communication, criticism-rejection and inductive form, so DoS have a direct effect on these variables, and an indirect effect on them through marital satisfaction. Although there is no previous empirical evidence about these relationships, these results are congruent with the postulates of Bowen [1] that state most differentiated people have better family relationships, including couple and parent-child relationships, and that the quality of couple’s relationship can affect the parenting through spillover and crossover processes [44]. Specifically, Bowen stated that people with higher levels of DoS do not tend to express their anxiety through couple conflict, emotional distancing from the partner, under or overfunctioning, or focusing on their children to calm their anxiety and solve their couple conflicts [11].

This research has some methodological limitations related to the characteristics of the sample, the online cross-sectional data collection and its interpretation. The sample could be larger and improve its gender imbalance. The collecting method prevent us from assessing the representativeness of the sample, and involve some possible self-report bias. Concerning the way of obtaining the data, all the instruments were self-reports, so that the answers may be biased, and only one informant was used, limiting our understanding of husband vs. wife or adolescent vs. parents perspectives. The data were collected in only one time point so longitudinal conclusions cannot be either achieved. We chose to use the DSS scale, instead of the Differentiation of Self Inventory-Revised (DSI-R) [3], which has been widely used in research. DSS allows us to evaluate all the theoretical dimensions of the Bowen’s construct, including the DO, while the Spanish validation of DSI-R [57] do not. This gives us a more complete perspective on the construct but limits the comparability of some of the results. Finally, the moderate magnitude of the relations invites us to be cautious when drawing conclusions.

Further research should complement the results obtained in this study collecting larger and representative samples of the Spanish population, and assessing both couple members or including adolescents’ evaluation of their parents’ parenting styles. Thereby, we can improve the empirical evidence about whether the assumptions of Bowen’s Family Systems Theory [1] in Spanish population are met, as well as increase the transcultural relevance of the theory.

Despite these limitations, these results are relevant since they support some of the assumptions of Bowen’s Family Systems Theory [1]. Furthermore, these results have several implications for couple and family therapy and psychoeducational practice with families during their children adolescence, because they point out the importance of the parents’ DoS and marital satisfaction in their parental practices. This offers therapists and counselors alternative approaches to promote better parenting practices, beyond direct psychoeducation concerning parenting styles. These intervention strategies may include couples counseling to improve marital satisfaction or individual therapy to strengthen parental DoS. For example, a therapist or a counselor could favor that parents adopt inductive styles with their children helping them to increase their DoS, understanding the developmental tasks of adolescents, managing their own emotional reactivity through mindfulness and relaxation or time-out techniques, and bonding with their children in an adjusted way, that is, avoiding the fused and dominant responses and the emotional cutoff [1, 11, 58]. In addition, the level of DoS of both members of a couple could be strengthened to improve their marital satisfaction, which in turn could increase warmth, communication and the use of a more inductive style [11]. Finally, the existing problems in the couple should be addressed, for example, through the Scheinkman’s Multi-level approach or the vulnerability cycle tool, so that the increase in marital satisfaction could enhance a more inductive parenting style with the children [1, 59, 60].

## Supporting information

S1 Data (SAV)
